# Diagnostic accuracy of Cone Beam Computed Tomography, conventional and digital radiographs in detecting interproximal caries

**Published:** 2015

**Authors:** Y Safi, N Shamloo Mahmoudi, MM Aghdasi, M Eslami Manouchehri, R Rahimian, S Valizadeh, Z Vasegh, Z Azizi

**Affiliations:** * Department of Oral and Maxillofacial Radiology, School of Dentistry, Shahid Beheshti University of Medical Sciences, Tehran, Iran,; ** Department of Oral and Maxillofacial Radiology, School of Dentistry, International branch of Shahid Beheshti University of Medical Sciences, Tehran, Iran

**Keywords:** Cone Beam CT, dental caries, diagnosis, digital, radiography

## Abstract

Statement of the problem: Presently, various imaging methods are available for the disclosure of proximal caries. Some recent studies have attempted to determine the diagnostic accuracy of available modalities, but they have shown variable results.

**Aim:** This study was carried out to recognize and examine the correctness of cone-beam computed tomography (CBCT), regular radiographs and the nondirect digital system in the disclosure of interproximal caries.

**Materials and Method:** In this observational tryout study, forty-two extracted non-cavitated, unrestored person molar and premolar teeth were placed in the blocks with proximal surfaces in touch. Then they were appraised by CBCT, formal radiographs and the nondirect digital system for the disclosure of interproximal caries. Four oral and maxillofacial radiologists used a 4-point scale to assess the pictures for the existence or absence of proximal caries. Caries depth was specified by histological examination. The gathered data were assessed by SPSS software using Weighted Kappa and Friedman test.

**Results:** Statistics demonstrated that the accuracy of the indirect digital system was somewhat better than conventional systems. The accuracy of the indirect digital system was better than cone beam system, and this difference was statistically significant.

**Conclusion:** The digital system was better than CBCT in the disclosure of proximal caries. The formal radiography fell in between the two other systems without a statistically significant deviation in detecting caries. Thus, CBCT is not advised to detect proximal caries because of the higher radiation dose.

## Introduction

Spotting of caries in the proximal faces of teeth has always been challenging [**1**]. Dental clinicians use visual examination and intraoral radiography to analyze caries [**2**]. Formal intraoral film radiography is a valid technique for the determination of proximal caries that cannot be readily identified by visual examination [**3**]. An alternative method is digital intraoral radiography [**4**,**5**]. Digital and formal radiography have the same accuracy for the disclosure of caries. But, they both lack in diagnostic accuracy for the identification of beginning proximal caries [**6**,**7**]. Intraoral radiographs are a 2-dimensional (2D) imaging technique that records 3-dimensional (3D) configurations. Some researches have appraised the use of 3D imaging method to evade the overlap of 3D anatomic configurations [**8**]. The cone-beam computed tomography (CBCT) method can be used in some dental regions such as implant treatment, craniofacial anomalies, endodontics, orthodontics, periodontology, as well as other dental conditions [**9**].

It has been clarified that without radiographic assessment, 25-42% of proximal caries may not be discovered by clinical examination [**10**,**11**]. Formal intraoral radiographs and photo stimulable phosphor (PSP) plates are the most frequently used image receptors [**12**,**13**]. Cone-beam computed tomography (CBCT) is a newly developed method that supplies three-dimensional information at a lower radiation dose than the formal CT [**14**]. The application of CBCT in dental condition has some benefits compared to formal imaging modalities, such as better image accuracy (in endodontic [**15**] and periodontic [**16**,**17**] application), fewer artifacts and higher cost-effectiveness [**18**].

The goal of this analyze was to contemplate the differences in the diagnostic correctness of different modalities and contradictions in earlier surveys, and the present study surveyed the in-vitro diagnostic capacity of radiographs, PSP sensors and CBCT in the discovering of proximal caries in posterior teeth.

## Materials and Method

The experimental trial study was conducted on 42 non-cavitated extracted human premolar and molar teeth. The clinical aspects of the tooth cover varied from perfect to discolored. Surfaces with fillings were dismissed. The teeth were stored in standard saline solution. The research method was confirmed by the Ethical Board of Shahid Beheshti University Dental School. Four teeth were installed in a row with the proximal surfaces in touch. Each row consisted of three test teeth and one non-test tooth in silicone blocks. The proximal caries was discovered by using radiographs, PSP and CBCT pictures. 

**Fig. 1:**
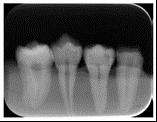
Digital Radiograph

**Fig. 2:**
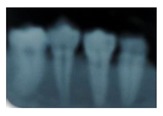
Conventional Radiograph

The specimens were radiographed by two intraoral modalities: 1) Digora-fmx with blue plates [Sordex, Helsinki, Finland] and 2) Kodak (Espeed) Insight film (size2) [Eastman Kodak Company, Rochester, NY, USA]. The digital pictures (**Fig. 1**) were taken at 70 kvp, 8 mA but the exposure time was reduced to 0.08 s. The focus-tooth and tooth-receptor distances were 32 and 2 cm, respectively. The software used for processing PSPs was Digora for windows 2.8. The conventional images (**Fig. 2**) were exposed with an X-ray unit operated at 70 kvp, 8 mA and exposure time of 0.16 s. The focus-tooth length was 32 cm and the tooth - receptor distance was 2 cm. Radiographs were processed after exposure, by using an automatic processing machine [Gendex, Clarimat, Milwaukee, WI, USA] and chemicals (X-ray Iran Company, Tehran, Iran) according to the producer's instruction.

**Fig. 3:**
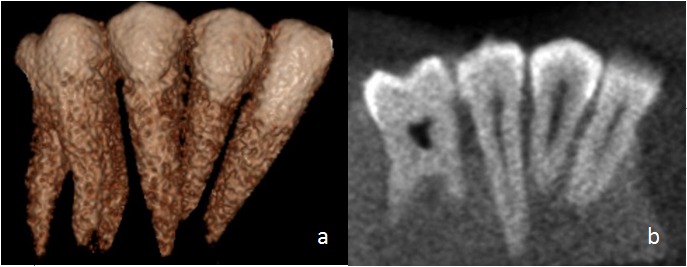
CBCT Image

The image of the teeth was also recorded by using CBCT system (**Fig. 3**) Newtom VGI [Quantitative Radiology, Verna, Italy] in selected FOV 6×6cm, high resolution at a fixed 110 kvp setting and auto-adjusted milliamperes. The blocks were scanned for 36 s.

In the use of intraoral modalities, a 12 mm acrylic plate was applied as fake soft tissue [**9**] between the tube and the mounted tooth. During the CBCT exposures, a water phantom [**3**] was placed around the blocks to resemble soft tissue. 

The pictures were evaluated separately by four expert oral radiologists. All the images were analyzed twice. The use of enhancement facilities to adjust contrast, brightness, and magnification was allowed. Parasagittal slices were reconstructed with 0.1 mm steps and 0.1 mm slice thickness in CBCT images for caries disclosure. Additionally, the observers could assess CBCT images in the axial, coronal or sagittal sections, in which the lesion was best discerned. 

The observers recorded caries by using a 4-point confidence rating scale:

0: Definitely no caries

1: Enamel caries (radiolucency in enamel)

2: Dentine caries (radiolucency in dentine)

3: Deep dentine caries (radiolucency extending to pulp)

The teeth were sectioned by Grand section unit [a Buehler Isomet low speed saw, Germany] in the mesiodistal direction into 0.1 mm thick sections. The sections were fixed on a glass slide. An experienced maxillofacial pathologist inspected the tooth sections by a light microscope (Eclipse E400, Nikon, Japan) and classified each tooth surface into one of four categories:

0: No deficiencies in the proximal outside 

1: Proximal defects in enamel

2: Proximal defects in the exterior half of the dentine 

3: Proximal defects in the interior half of the dentine 

The variations among the witnesses were assessed with Friedman examination and differences in sensitivity and specificity were analyzed by using the Weighted Kappa examination. The SPSS v.16 software was done in statistical analysis. 

## Results

**Chart 1:**
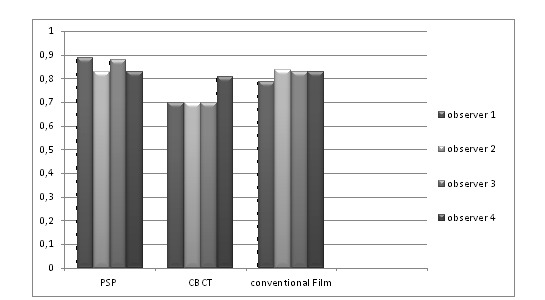
Diagnostic accuracy for each method for observers on mesial surface

**Chart 2:**
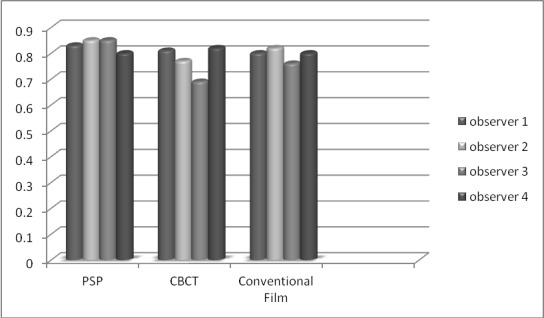
Diagnostic accuracy for each method for observers on distal surface

The histological tests showed that out of 84 proximal outsides, 54 (64%) were healthy, 11 (13%) had enamel caries, 15 (18%) had caries in the exterior half of the dentin and 4 (5%) had dentine caries, reaching the interior half of the dentin. Four witnesses individually compared the caries symptomatic exactness of three modalities [CBCT, digital radiography (PSP) and film radiography]. **Chart 1** and **2** show the diagnostic accuracy of each method for observers on mesial and distal surfaces, respectively.

The specificity value of PSP was 94.9%; which means that most surfaces were assessed caries free by PSP (the specificity value of the radiographs and CBCT were 95.4% and 83.8%, respectively) and its positive predictive rate was 50%.The enamel caries sensitivity value was 56.8% for PSP, 31.8% for conventional film radiography and 30.2%, for CBCT; whereas the negative predictive value of PSP, CBCT and conventional radiography was 89.9% 83.8% and 82.4%, respectively. 

No differences were found for any parameter between PSP and radiographs, and between radiographs and CBCT. **Table 1**-**3** present obtained parameters of each method by observers.

**Table 1:** Conventional Technique parameters

Conventional Technique	Radiolucency					
Golden Standard		None	In enamel	In the inner half of dentin	In the outer half of dentin	Total
Caries Lesions	None	95.4%(206)	1.9% (4)	2.3% (5)	0.5% (1)	100%(216)
	In enamel	61.4%(27)	31.8%(14)	6.8% (3)	0% (0)	100% (44)
	In the inner half of dentin	30.8%(16)	9.67% (5)	57.7%(30)	1.9% (1)	100% (52)
	In the outer half of dentin	4.2% (1)	0% (0)	66.7%(16)	29.2% (7)	100% (24)
	Total	74.4% (250)	6.8% (23)	6.1% (54)	2.7% (9)	100% (336)

There was no statistically important variation among witnesses but the outcomes showed variations between the various methods of caries discovery. P<0.05 was considered statistically important. 

**Table 2:** PSP Technique parameters

PSP	Radiolucency					
Golden Standard		None	In enamel	In the inner half of dentin	In the outer half of dentin	Total
Caries Lesions	None	94.9%(205)	4.6% (10)	0.5% (1)	0% (0)	100%(216)
	In enamel	40.9%(18)	56.8%(25)	2.3% (1)	0% (0)	100% (44)
	In the inner half of dentin	9.6%(5)	28.8% (15)	59.6%(31)	1.9% (1)	100% (52)
	In the outer half of dentin	0% (0)	0% (0)	37.5%(9)	62.5% (7)	100% (24)
	Total	67.9% (228)	14.9% (50)	12.5% (42)	4.8% (16)	100% (336)
*PSP: Photostimulable Phosphor Plate*						

**Table 3:** CBCT Technique parameters

CBCT	Radiolucency					
Golden Standard		None	In enamel	In the inner half of dentin	In the outer half of dentin	Total
Caries Lesions	None	83.8%(181)	7.9% (17)	8.3% (18)	0% (0)	100%(216)
	In enamel	58.1%(25)	30.2%(13)	11.7% (6)	0% (0)	100% (44)
	In the inner half of dentin	17.5%(9)	17.5%(9)	61%(32)	4% (2)	100% (52)
	In the outer half of dentin	4.2% (1)	16.7% (4)	45.8%(11)	33.3% (8)	100% (24)
	Total	64.2% (216)	13% (43)	19.8% (66)	3% (10)	100% (336)
*CBCT: Cone Beam Computed Tomography*						

## Discussion

This examination was done on non-cavitated teeth to discover and analyze the accuracy of cone beam computed tomography, standard radiograph and the indirect digital system in identifying interproximal caries. CBCT is a recently extended dental imaging method with unclear characteristic possibilities for some duties. This novel imaging modality may be interesting to clinicians for caries discovery goals. To verify the accuracy of a novel diagnostic modality, it has to be examined and compared with the possible well-documented imaging methods. In the present in vitro research, proximal caries discovery correctness was assessed by one of the well-known CBCT imaging methods. Additionally, radiographs and the PSP were involved in this study as standard detectors for the measurement of the extent of proximal caries. 

Our research was done on non-cavitated teeth with small clinical demineralization. The sensitivity values for the discovery of incipient enamel injuries in proximal covers were 56.8%, 31.8% and 30.2% for PSP, conventional radiographs and CBCT modalities, respectively. Considering the challenge in identifying early enamel defects, normal radiographs and CBCT were both comparable in accuracy while their accuracy was weaker than that of a PSP. The use of image improvement tools may justify the higher accuracy of digital systems. 

Because of the simplicity of discovering deficiencies in the exterior half of the dentin, all surveyed methods showed the same results. The sensitivity value of PSP in identifying radiolucency in the interior half of dentin was 62.5%; whereas this rate was 29.2% and 33.3% for the radiographs and CBCT, respectively. The higher sensitivity of digital systems versus radiographs was attributed to using more regulation of illumination and contrast. CBCT pictures have a lower spatial resolution [**19**], which appears in lower characteristic accuracy.

Based on the outcomes of the current research, no statistically important distinction in non-cavitated proximal caries discovery accuracy was observed within the PSP and radiographs (p>0.05) or CBCT and radiographs (p>0.05). But, a statistically important difference in the diagnostic accuracy among CBCT and PSP (p<0.05) was determined. The results were by initial studies comparing proximal caries discovery in normal radiographs, digital, and CBCT pictures [**20**-**22**]. The radiographs, digital, and CBCT imaging methods used in those investigations were similar to the ones examined herein. 

Although some of researches have discovered that the CBCT is better for the analysis of dentin caries [**23**,**24**], it should be held in mind that CBCT pictures are very dependable in recognizing the presence of a cavity in a proximal tooth [**25**]. This study discovered no advantage of CBCT over radiographs or PSP for the disclosure of caries surfaces. Based on Akdeniz et al. research, some types of CBCT methods such as Accuitomo are effective means for the analyze and monitoring of proximal caries [**23**]. In opposition to Akdeniz et al. research, our research found no variations between CBCT and normal intraoral radiographs and even Newtom CBCT method had a significantly lower diagnostic accuracy than the PSP. The variations in these studies may be due to the type of company tools. 

Researches have informed that the CBCT had a greater radiation dose compared to a normal intraoral radiograph [**26**]. Consequently, taking a CBCT only for the discovery of proximal caries is not suggested. 

Other studies have shown that the discovery correctness of proximal caries in CBCT, digital radiography and traditional radiography are alike [**3**,**27**,**28**]. Variations among these studies may be described by some factors. Firstly, various groups of witnesses were used (Zhi-ling Zhang et al. used students as witnesses [**3**]). Secondly, the observers in this research used the picture improvement equipment as they pleased and thirdly, the CBCT systems employed in these studies were not the identical. 

Some false positive diagnoses happened with the radiographs and Digora-fmx (4.7% and 5.1%, respectively) than with the CBCT (16.2%). No important variations were seen among radiographs and PSP. according to these outcomes, digital intraoral methods are suggested over radiographs because of their weaker degrees of radiation dose. 

Some investigations have assessed the diagnostic potential of CBCT systems. Menegalet al. [**29**,**30**] used the Accuitomo CBCT system for evaluating periodontal and peri-implant defects in contrast with intraoral radiography, panoramic radiography and CT. Moreover, Misch et al. [**31**] assessed interproximal periodontal defects by using the i-CAT CBCT, intraoral F-speed film, and CT and explained that CBCT was better than the other systems. 

Regarding these results, the Newtom CBCT system has a lower characteristic accuracy than the intraoral modalities for caries discovery. Due to high patient dose, it was not logical to do this research on human specimens. Restored teeth were rejected because of metal artifacts result from the metallic restoration that may compromise picture quality and diagnostic accuracy.

## Conclusion

In this study, tried to analyze the diagnostic correctness of CBCT, traditional radiography, and PSP for the discovering of proximal caries, differences between these modalities were not meaningful with no benefits of CBCT imaging. Therefore, CBCT is not recommended to discover proximal caries because of the greater radiation dose. 

**Acknowledgment**

This report was based on a postgraduate thesis by Dr. Rahimian, which was successfully performed under the supervision of Dr. Safi with the close cooperation of the Pathology Department of the Dental School of Shahid Beheshti University of Medical Sciences. The authors express their sincere appreciations to all observers who evaluated the test radiographs. 
